# Prediction of Antibiotic Resistance Evolution by Growth Measurement of All Proximal Mutants of Beta-Lactamase

**DOI:** 10.1093/molbev/msac086

**Published:** 2022-04-29

**Authors:** Siyuan Feng, Zhuoxing Wu, Wanfei Liang, Xin Zhang, Xiujuan Cai, Jiachen Li, Lujie Liang, Daixi Lin, Nicole Stoesser, Yohei Doi, Lan-lan Zhong, Yan Liu, Yong Xia, Min Dai, Liyan Zhang, Xiaoshu Chen, Jian-Rong Yang, Guo-bao Tian

**Affiliations:** Department of Microbiology, Zhongshan School of Medicine, Sun Yat-sen University, Guangzhou 510080, China; Key Laboratory of Tropical Diseases Control, Sun Yat-sen University, Ministry of Education, Guangzhou 510080, China; Department of Biomedical Informatics, Zhongshan School of Medicine, Sun Yat-sen University, Guangzhou 510080, China; Department of Microbiology, Zhongshan School of Medicine, Sun Yat-sen University, Guangzhou 510080, China; Key Laboratory of Tropical Diseases Control, Sun Yat-sen University, Ministry of Education, Guangzhou 510080, China; Department of Biomedical Informatics, Zhongshan School of Medicine, Sun Yat-sen University, Guangzhou 510080, China; Department of Genetics and Cellular Biology, Zhongshan School of Medicine, Sun Yat-sen University, Guangzhou 510080, China; Department of Microbiology, Zhongshan School of Medicine, Sun Yat-sen University, Guangzhou 510080, China; Key Laboratory of Tropical Diseases Control, Sun Yat-sen University, Ministry of Education, Guangzhou 510080, China; Department of Microbiology, Zhongshan School of Medicine, Sun Yat-sen University, Guangzhou 510080, China; Key Laboratory of Tropical Diseases Control, Sun Yat-sen University, Ministry of Education, Guangzhou 510080, China; Department of Microbiology, Zhongshan School of Medicine, Sun Yat-sen University, Guangzhou 510080, China; Key Laboratory of Tropical Diseases Control, Sun Yat-sen University, Ministry of Education, Guangzhou 510080, China; Modernising Medical Microbiology, Nuffield Department of Medicine, University of Oxford, Oxford, United Kingdom; Division of Infectious Diseases, University of Pittsburgh School of Medicine, Pittsburgh 15261, PA, USA; Department of Microbiology, Fujita Health University School of Medicine, Aichi 470-1192, Japan; Department of Infectious Diseases, Fujita Health University School of Medicine, Aichi 470-1192, Japan; Department of Microbiology, Zhongshan School of Medicine, Sun Yat-sen University, Guangzhou 510080, China; Key Laboratory of Tropical Diseases Control, Sun Yat-sen University, Ministry of Education, Guangzhou 510080, China; Clinical Laboratory, Fifth Affiliated Hospital, Sun Yat-sen University, Zhuhai 519000, China; Department of Clinical Laboratory Medicine, Third Affiliated Hospital of Guangzhou Medical University, Guangzhou, China; School of Laboratory Medicine, Chengdu Medical College, Chengdu 610500, China; Department of Clinical Laboratory, Guangdong Provincial People’s Hospital/Guangdong Academy of Medical Sciences, Guangzhou, Guangdong 510080, China; Department of Genetics and Cellular Biology, Zhongshan School of Medicine, Sun Yat-sen University, Guangzhou 510080, China; Key Laboratory of Tropical Diseases Control, Sun Yat-sen University, Ministry of Education, Guangzhou 510080, China; Department of Biomedical Informatics, Zhongshan School of Medicine, Sun Yat-sen University, Guangzhou 510080, China; RNA Biomedical Institute, Sun Yat-Sen Memorial Hospital, Sun Yat-sen University, Guangzhou 510120, China; Department of Microbiology, Zhongshan School of Medicine, Sun Yat-sen University, Guangzhou 510080, China; Key Laboratory of Tropical Diseases Control, Sun Yat-sen University, Ministry of Education, Guangzhou 510080, China; School of Medicine, Xizang Minzu University, Xianyang, Shaanxi 712082, China

**Keywords:** antibiotic resistance, prediction model, β-lactamase, evolutionary trajectories, high-throughput sequencing

## Abstract

The antibiotic resistance crisis continues to threaten human health. Better predictions of the evolution of antibiotic resistance genes could contribute to the design of more sustainable treatment strategies. However, comprehensive prediction of antibiotic resistance gene evolution via laboratory approaches remains challenging. By combining site-specific integration and high-throughput sequencing, we quantified relative growth under the respective selection of cefotaxime or ceftazidime selection in ∼23,000 *Escherichia coli* MG1655 strains that each carried a unique, single-copy variant of the extended-spectrum β-lactamase gene *bla*_CTX-M-14_ at the chromosomal *att* HK022 site. Significant synergistic pleiotropy was observed within four subgenic regions, suggesting key regions for the evolution of resistance to both antibiotics. Moreover, we propose PEAR^P^ and PEAR^R^, two deep-learning models with strong clinical correlations, for the prospective and retrospective prediction of *bla*_CTX-M-14_ evolution, respectively. Single to quintuple mutations of *bla*_CTX-M-14_ predicted to confer resistance by PEAR^P^ were significantly enriched among the clinical isolates harboring *bla*_CTX-M-14_ variants, and the PEAR^R^ scores matched the minimal inhibitory concentrations obtained for the 31 intermediates in all hypothetical trajectories. Altogether, we conclude that the measurement of local fitness landscape enables prediction of the evolutionary trajectories of antibiotic resistance genes, which could be useful for a broad range of clinical applications, from resistance prediction to designing novel treatment strategies.

## Introduction

Bacterial antimicrobial resistance (AMR) is a serious global public health concern associated with significant clinical, economic, and social impacts. A major contributor to the dissemination of clinically relevant AMR genes is plasmid-mediated horizontal transfer, in which mobilizable plasmids facilitate the intra- and interspecies transmission of AMR genes. Additionally, horizontally transferred genes can produce allelic variants that confer increased resistance to more antibiotics. For example, *bla*_TEM-1_, the first plasmid-encoded β-lactamase ever described (Datta and Kontomichalou 1965) hydrolyzes only broad-spectrum penicillins. However, *bla*_TEM-43_, a variant of *bla*_TEM-1_ with only three amino acid substitutions (E102K, R162H, and M180T), confers resistance to all penicillins and cephalosporins (Yang et al. 1998). While many strategies have been considered for controlling AMR, there remains an urgent need to better understand and predict how AMR genes evolve.

The prediction of AMR evolution, particularly among horizontally transferred genes, may contribute to the strategic design of appropriate treatments (Raymond et al. 2002; Yeh et al. 2009; Lee et al. 2013; Munck et al. 2014). There are two main approaches that have been used to predict the evolution of bacterial AMR. On the one hand, clinical studies have focused on the identification of gene variants (the clinical isolates) and evolutionary trajectory prediction by characterizing individual and multiple substitutions (Novais et al. 2010; Yang et al. 2020). On the other hand, experimental studies have focused on the evolutionary theory using *in vitro* model systems to investigate genotype–phenotype relationship (Bratulic et al. 2015; Stiffler et al. 2015; Figliuzzi et al. 2016; Bratulic et al. 2017), with less emphasis on AMR genes or variants with greater clinical significance, such as the rapidly spreading CTX-M beta-lactamases (An et al. 2016; Gladstone et al. 2021).

Randomized mutagenesis through approaches such as error-prone polymerase chain reaction (PCR) is a common in vitro method of studying plasmid-mediated AMR genes (Barlow and Hall 2002; Bratulic et al. 2015; Rosenkilde et al. 2019). While large numbers of single substitutions found in naturally occurring AMR genes can be recovered by error-prone PCR, the clinical application of these in vitro evolutionary studies has been limited. In part, this is because they have covered a relatively low proportion of single-nucleotide substitutions (hereafter, single mutations), which could have contributed to sequential AMR development. To comprehensively evaluate the mutational effect and predict the likely trajectory of resistance in clinical settings, studies should analyze all possible mutations. In this regard, it is becoming increasingly important to develop novel models with more comprehensive coverage (>90%) of single mutations.

Here, we presented a novel experimental framework to study AMR gene evolution by examining *bla*_CTX-M-14_, which is highly relevant to the rapid emergence of multidrug resistance in Enterobacterales, a globally common cause of bacterial infection(Hamamoto et al. 2016; Bevan et al. 2017; Hayashi et al. 2018). CTX-M-14 primarily confers resistance to cefotaxime, but can mutate to expand its spectrum of hydrolysis to ceftazidime, another commonly used third-generation cephalosporin. Using site-specific integration, we generated ∼200,000 *Escherichia coli* MG1655 strains, each carrying a variant of the single-copy *bla*_CTX-M-14_ gene at the chromosomal *att* HK022 site. We determined, with high throughput and accuracy, the relative growth of ∼23,000 strains under cefotaxime and ceftazidime selection. Our data covered >90% of single mutations in the *bla*_CTX-M-14_ gene, and revealed key regions for the evolution of cross-resistance to cefotaxime and ceftazidime. Utilizing this unprecedented dataset, we developed a novel deep-learning model for *P*redicting *E*volution of *A*ntibiotic *R*esistance (PEAR), and applied it for the prospective (PEAR^P^) and retrospective (PEAR^R^) prediction of *bla*_CTX-M-14_ evolution. Comparison with *bla*_CTX-M_ variants identified in clinical bacterial isolates suggested that PEAR^P^ and PEAR^R^ are powerful tools for assessing antibiotic resistance risk and performing the origin-tracing of novel *bla*_CTX-M_ variants, respectively. Collectively, our results provide novel insights into the evolution of AMR, and our approach can be used to support the strategic design and evaluation of antibiotic treatments (either singly or in combination) for different drug-resistant bacteria.

## Results

### Massively Parallel Measurement of the Relative Growth of all Proximal Mutants of *bla*_CTX-M-14_ Under Antibiotic Selection

**Fig. 1.**
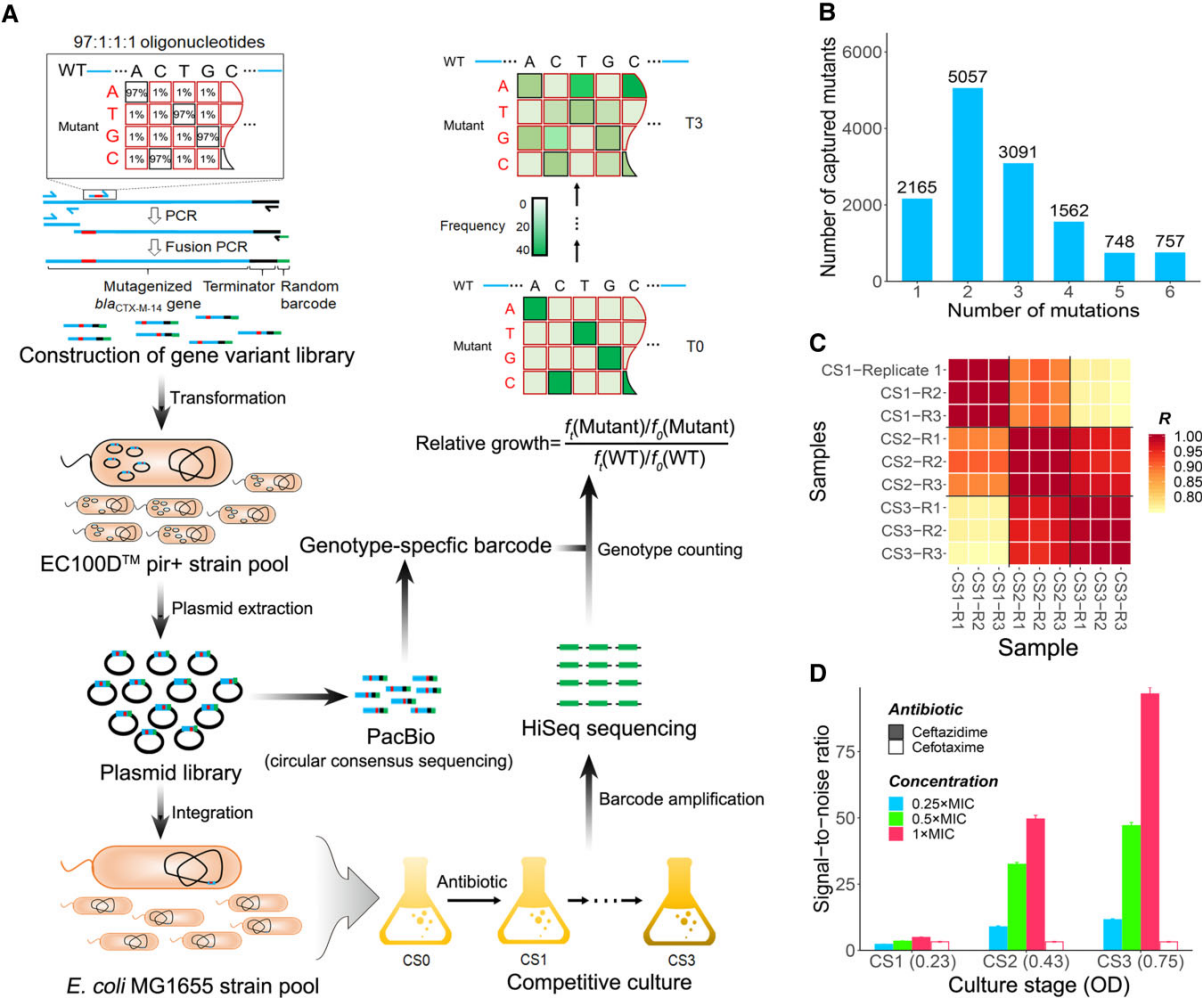
Determining the fitness landscape of the *bla*_CTX-M-14_ gene. (*A*) Illustration of the experimental workflow for assessing the fitness landscape of CTX-M-14. The *bla*_CTX-M-14_ variant library was generated using “doped” oligonucleotides (3% per-site mutation rate). Then, the variant library was cloned into the integrated pOSIP-KH plasmid to create the *pir*^+^ strain pool. The plasmid library was sequenced with the PacBio Sequel System after plasmid extraction in order to determine the correspondence between genotypes and barcodes. The plasmid library was then integrated into the *Escherichia coli* MG1655 genome. Competition experiments were conducted in LB liquid medium containing cefotaxime or ceftazidime. After barcode amplification, Illumina HiSeq sequencing was used to obtain the frequency of mutant genotype *f* (Mutant) or wild-type *f* (WT). The relative growth of each genotype was evaluated as the increase in frequency under antibiotic selection relative to wild-type *bla*_CTX-M-14_. (*B*) Numbers of variants with 1–6 single-nucleotide mutations whose relative growth was determined in our experimental pipeline. (*C*) Mutual comparison of genotype frequencies between biological replicates at CS1, CS2, and CS3 in the presence of 1 × MIC ceftazidime. At each culture stage, three biological replicates are represented, for example, CS1 repeat 1 (CS1-R1). The color scale represents *R* (Pearson's correlation coefficient) between samples (see also supplementary fig. S2*C*, Supplementary Material online). (*D*) SNR of the relative growth, estimated by variation among barcodes of the same genotype (see Materials and Methods). CS1–CS3 (*x* axis) represent three culture stages, with the corresponding OD_600_ values listed within parentheses. Error bars represent standard error.

The “fitness landscape” depicts the relationship between genotype and bacterial fitness (i.e., growth rate); it is therefore a major determinant of AMR gene evolution and the main focus of our study. CTX-M-14 comprises an N-terminal signal peptide of 26 amino acids and a mature β-lactamase of 263 amino acids. We aimed to assess the local fitness landscape of a mature β-lactamase, that is, the fitness of all possible single mutations and a fraction of multi-mutation alleles of *bla*_CTX-M-14_. To this end, we used “doped” oligonucleotides (3% per-site mutation rate) to generate a *bla*_CTX-M-14_ variant library, in which each variant included a unique 20-nt barcode in the downstream region of the *rrnB* terminator to avoid interference with CTX-M-14 function (fig. 1*A*). Using these variants, we constructed a library of ∼200,000 EC100D^TM^  *pir*^+^ colonies. We used the PacBio Sequel System to determine both the 789-bp mature *bla*_CTX-M-14_ sequence and the 128-bp barcode + terminator sequence for a single molecule. To improve accuracy, circular consensus sequencing (CCS) was employed, and only sequencing wells with at least five subreads were included (supplementary fig. S1, Supplementary Material online, see Materials and Methods). Finally, this library included 1,824 single mutants with specific barcodes, and the accuracy of the sequences was ∼92.9% according to Sanger sequencing of randomly picked clones (supplementary table S1, Supplementary Material online). To include all the single mutants (789 × 3 = 2,367) of the mature *bla*_CTX-M-14_ sequence, the remaining 543 single mutants were manually constructed and then added to the plasmid library (see Materials and Methods).

Next, the plasmid library was integrated into the chromosomal *att* HK022 site of *E. coli* MG1655. Competition experiments were performed under selection by either cefotaxime or ceftazidime. Genotype frequencies were highly correlated between two technical repeats at culture stage zero (CS0) (Pearson's correlation, *R* ≥ 0.9999) (supplementary fig. S2*A*, S2*B*, Supplementary Material online). To accurately estimate the relative growth of the variants, ∼23,000 genotypes with read counts ≥100 at CS0 were included for further analysis. Among these genotypes, 2,165/2,367 (91.5%) possible single-point mutations were represented (fig. 1*B*). Genotype frequencies were highly correlated between biological replicates at CS1, CS2, and CS3, and the mean Pearson's *R* of all replicate pairs was 0.9977 (fig. 1*C* and supplementary fig. S2*C*, Supplementary Material online). By comparing the frequency change of a genotype during the competition with that of the wild-type, we estimated the growth of each variant relative to wild-type CTX-M-14 (fig. 1*A*. See also Materials and Methods). This measure of “relative growth” therefore captures the degree of antibiotic resistance conferred by each CTX-M-14 mutant relative to that of wild-type CTX-M-14, and the relative growths of all captured genotypes collectively constitute the fitness landscape of CTX-M-14. We found that the relative growth estimated from different biological replicates showed significant correlations with one another, and these correlations strengthened as we considered mutations with larger effects (supplementary fig. S2*D*, Supplementary Material), indicating that mutants with relative growth ≥2 are highly reproducible among replicates. Moreover, the signal-to-noise ratio (SNR) calculated by comparing different barcodes of the same genotype (see Materials and Methods) revealed that the measured relative growth was reliable and, as expected, became more accurate as the competition experiment continued (fig. 1*D* and supplementary fig. S2*E*, Supplementary Material online).

### The Local Fitness Landscape of *bla*_CTX-M-14_ in *E. coli*

**Fig. 2.**
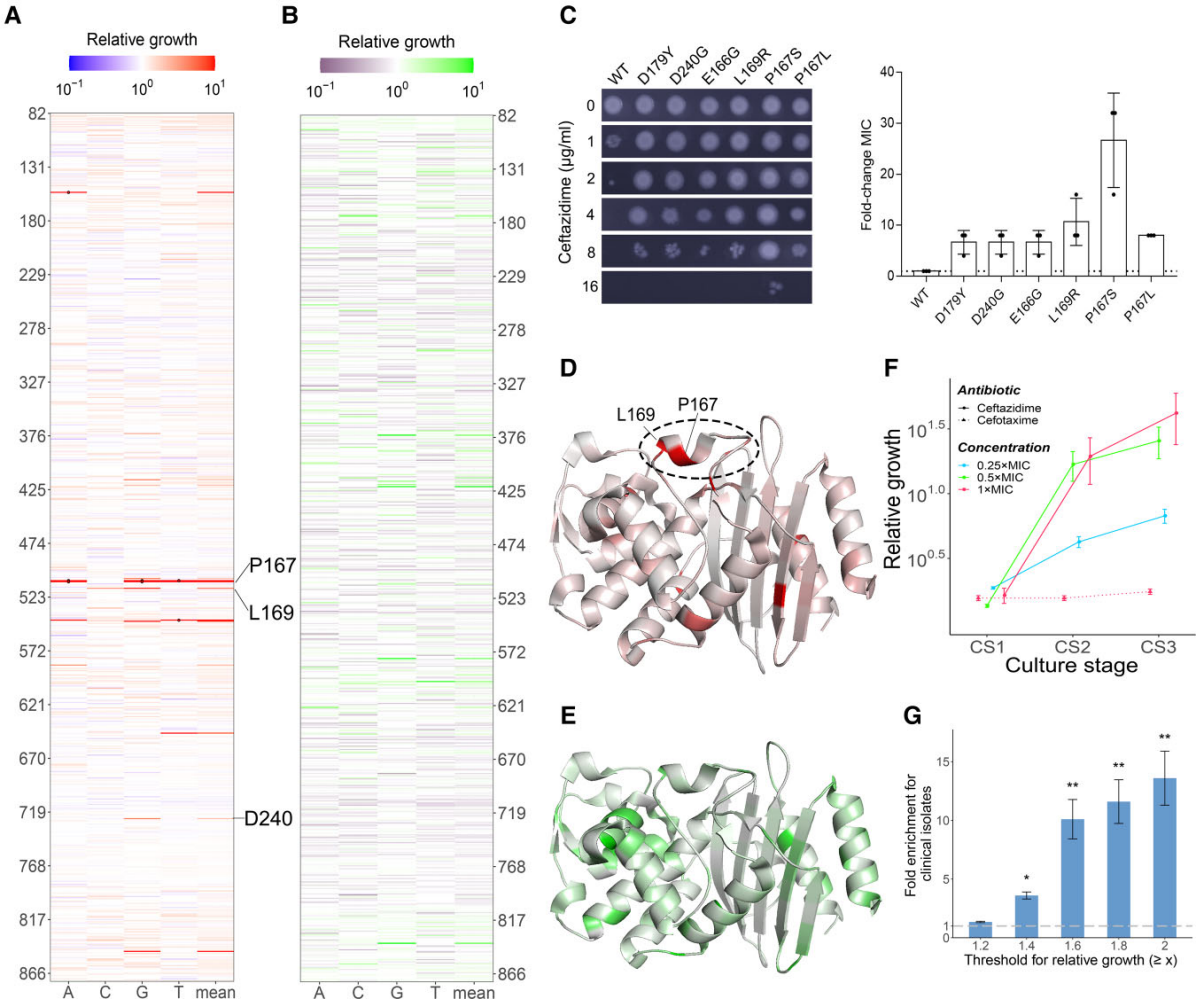
Fitness effects of all single-nucleotide mutations in CTX-M-14 under antibiotic selection. (*A* and *B*) Fitness landscape of *bla*_CTX-M-14_ in the presence of 1 × MIC ceftazidime (*A*) or cefotaxime (*B*) at CS3. Each tile represents a variant with one single-nucleotide mutation (*x* axis) at one specific position (*y* axis), whose relative growth is indicated by the color of the tile scaled according to the corresponding color scale bar on top. In addition, mutants with relative growth >100 are marked with black dots. (C) Functional assay of six mutations in CTX-M-14 essential for ceftazidime resistance. A representative result from three independent experiments is presented. The bars and error bars respectively represent the mean and the standard deviation. (*D* and *E*) For the relative growth measured in the presence of 1 × MIC ceftazidime (*D*) or cefotaxime (*E*), the maximum value of relative growth associated with the nine single-nucleotide substitutions at an amino acid position is indicated by colors overlaid on the three-dimensional structure of CTX-M-14 (PDB ID: 6D7H). The omega loop of CTX-M-14 required for antibiotic hydrolysis is highlighted with an oval. (*F*) The average relative growth of all beneficial (defined as relative growth ≥1.2 at CS3) mutants were increased in a time-dependent manner in the presence of ceftazidime and cefotaxime. The error bar represents the standard deviation. (*G*) Enrichment of mutations found in clinical isolates among CTX-M-14 single-nucleotide mutants with elevated relative growth in 1 × MIC ceftazidime at CS3, relative to all but nonsense mutants. The enrichment (*y* axis) strengthens as the threshold for relative growth (*x* axis) increases. The *P* values of hypergeometric tests are indicated. *P < 0.01, **P < 0.001. The error bar represents the standard error of mean.

We first focused on the relative growth of single-nucleotide *bla*_CTX-M-14_ mutants (figs. 2*A* and *B*). A few mutations dramatically increased relative growth in ceftazidime (marked by black dots in fig. 2*A*; supplementary table S2, Supplementary Material online). These mutations correspond to some well-known ceftazidime resistance mutations, such as P167S (Patel et al. 2017) and D240G (Bonnet et al. 2003). In particular, almost all nonsynonymous single-nucleotide mutants (S, L, H, A, T) of P167 exhibit 100 to over 4,700-fold increases in relative growth in the presence of ceftazidime compared to that of wild-type CTX-M-14, strongly suggesting that proline at position 167 constrained the protein flexibility and therefore the hydrolytic activity of the enzyme (Poirel et al. 2001; Kimura et al. 2004). In turn, this observation suggested that any amino acid substitution at P167 may increase resistance to ceftazidime (fig. 2*C*), which was indeed demonstrated by the minimal inhibitory concentrations (MICs) of all 19 amino acid replacements of P167 (supplementary table S3, Supplementary Material online). In contrast to the results observed with ceftazidime, all single-nucleotide mutations had only mild effects (up to 18.8-fold in cefotaxime, compared to up to 4766-fold in ceftazidime) on relative growth in cefotaxime (1 × MIC) (fig. 2*B*; supplementary table S4, Supplementary Material online). This observation is understandable as CTX-M-14 had already evolved for some time under cefotaxime selection. In addition, when the maximum relative growth observed for the substitutions of each amino acid was overlaid on the three-dimensional structure of the wild-type CTX-M-14 enzyme (fig. 2*D* and *E*), the structural basis for the mutational effects became immediately apparent. For example, both P167 and L169, which had very strong mutational effects on relative growth in the presence of ceftazidime, face the active site where hydrolysis occurs (fig. 2*D*). Furthermore, the three top regions with the most significant enrichment of growth-enhancing mutations (supplementary fig. S3*A*, Supplementary Material online. See also Materials and Methods) were also compatible their known functional roles. Specifically, region 1 consisting of C69, N104, D163-A172, D179, and G238 (supplementary fig. S3*B*, Supplementary Material online), region 2 consisting of V29, L48, G224-P226, V260, and A280-G289 (supplementary fig. S3*C*, Supplementary Material online), and region 3 consisting of L48-V57 (supplementary fig. S3*D*, Supplementary Material online) are located in the active site, H11 helix, and B1-B2 β-strand, respectively (supplementary fig. S3, Supplementary Material online).

To further corroborate the accuracy of our data, we compared the relative growths estimated for different culture stages and under different concentration of ceftazidime. We found that the average relative growth of all mutants was higher at later culture stages during competitive culture and in the presence of lower concentrations of ceftazidime (fig. 2*F*), which suggested that resistant mutants generally displayed consistent growth advantages as competition continued or under lower antibiotic stress. We also noticed an expected decrease of relative growth for mutants carrying nonsense mutations (supplementary fig. S4*A*, Supplementary Material online). Moreover, the codon adaptation index (CAI) of synonymous mutants was found to be positively correlated with its relative growth (Spearman's *ρ* = 0.12, *P* = 0.0002; supplementary fig. S4*B*, Supplementary Material online), which was consistent with the known role of codon usage bias in translational regulation (Sharp and Li 1987; Plotkin and Kudla 2011; Chen et al. 2020). Importantly, mutants with increased relative growth were apparently enriched for mutations that are also known to be common clinical variants of CTX-M-14 (fig. 2*G*, see Materials and Methods), indicating the potential biomedical applications of our measured fitness landscape.

### Genetic Interactions and Genetic/Environmental Interactions of CTX-M-14

**Fig. 3.**
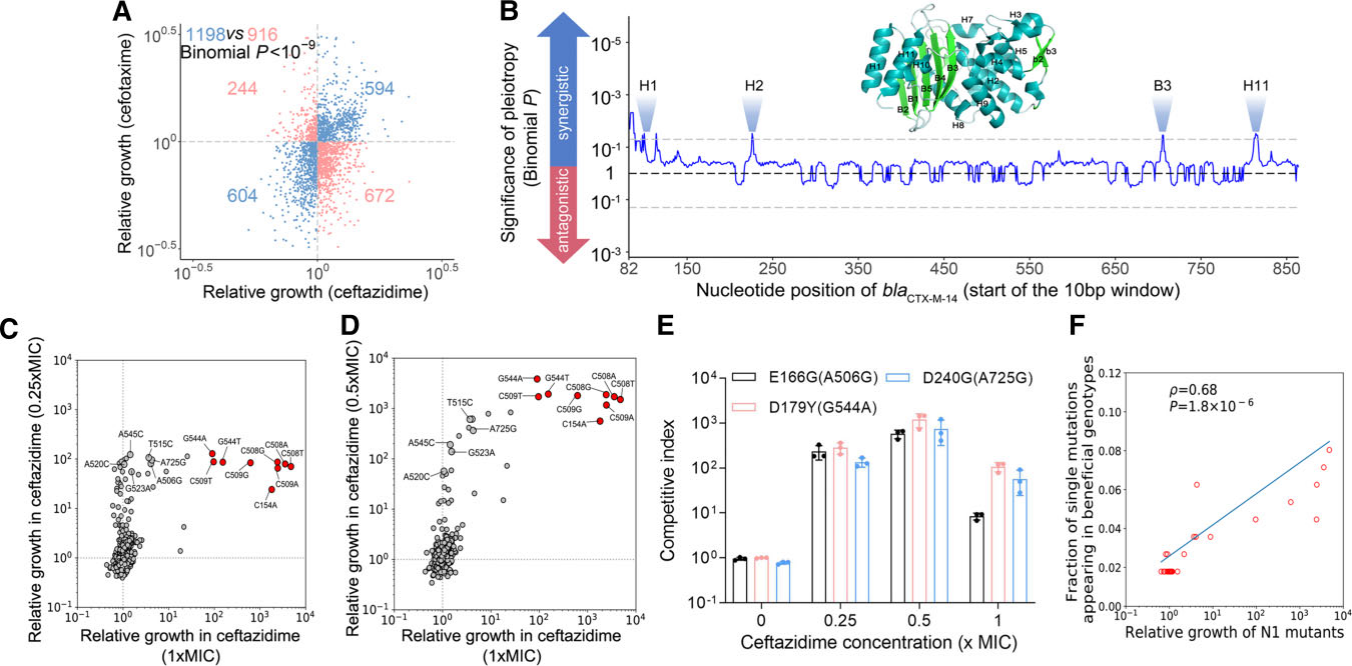
Pleiotropy and epistasis of CTX-M-14. (*A*) Pleiotropy of CTX-M-14 mutations for relative growth in ceftazidime and cefotaxime (1 × MIC at CS3). Blue dots represent synergistic pleiotropy; red dots represent antagonistic pleiotropy. Number of dots within each quadrant is respectively indicated. The binomial *P* value against the null expectation of equal chances of antagonistic/synergistic pleiotropy is shown. (*B*) Pleiotropy of subgenic regions within *bla*_CTX-M-14_ on relative growth in ceftazidime and cefotaxime (1 × MIC at CS3). Sliding windows of 10 bp (i.e., position 82 denotes the region between 82 and 91, and 83 denotes between 83 and 92, etc.) were used to analyze the whole gene encoding for the mature β-lactamase. Two-tailed binomial *P* values corrected for multiple testing (by the Benjamini–Hochberg procedure) are shown for each 10-bp window (*y* axis). The black dashed line indicates *P* = 1, and the gray dashed line indicates *P* = 0.05. (*C* and *D*) Comparison of relative growth for all single-nucleotide mutants under various concentrations of ceftazidime at CS3. Several mutations previously reported to confer an “extended-spectrum” phenotype in the CTX-M family among clinical isolates are shown in red with annotations describing the mutations. (*E*) The competitive indexes of three *Escherichia*  *coli* MG1655 strains harboring different CTX-M-14 variants. sfGFP-labeled *E. coli* MG1655 (pOSIP-KH-CTX-M-14_WT_) was mixed with mCherry-labeled *E. coli* MG1655 (pOSIP-KH-CTX-M-14_mutant_) in a 1 : 1 ratio, and the bacteria were co-cultured in presence of the designated concentration of ceftazidime (*x* axis). The mixed population in the log phase of growth was analyzed by flow cytometry. The competitive index is calculated as (*M*_t_/*M*_0_)/(WT_t_/WT_0_), whereas M_t_ and M_0_ are, respectively, the frequency of mCherry-labeled mutant cells at the beginning and end of the co-culture, and WT_t_ and WT_0_ are, respectively, the frequency of sfGFP-labeled WT cells at the beginning and end of the co-culture. Error bars represent the standard error of the mean (*n* = 3). (*F*) Single-nucleotide mutations that conferred higher resistance (*x* axis) tended to appear more often in beneficial variants (relative growth ≥2, including variants with single or multiple mutations). N1 mutants that appeared only once were not considered. N1 mutants are variants with one single-nucleotide substitution. ρ is Spearman's rank correlation coefficient, and the related *P* value and regression line (blue) are shown.

One primary factor governing the sequence evolution of CTX-M-14 is the environment (Palmer et al. 2015), as mutations could be pleiotropic (i.e., different phenotypic effects in different environments). In the realm of antibiotic resistance, it is also of mounting importance to assess the cross-resistance of different antibiotics caused by the same mutation. In this regard, comparisons between landscapes of relative growth measured in the presence of cefotaxime and ceftazidime (1 × MIC) (fig. 3*A*) showed that the majority of single-nucleotide mutations displayed synergistic pleiotropy (i.e., become more resistant or susceptible to both antibiotics; blue dots in fig. 3*A*), whereas only a minority of these mutations displayed antagonistic pleiotropy (i.e., become more resistant to one antibiotic and more susceptible to the other antibiotic; red dots in fig. 3*A*) (binomial *P* < 10^−9^). Such bias for synergistic pleiotropy was further enhanced when only mutations with larger absolute effect sizes were considered (supplementary fig. S5, Supplementary Material online). This observation was explainable by the similar mechanisms of action of cefotaxime and ceftazidime and the fact that they are members of the same antibiotic class (the third-generation cephalosporins). Moreover, a closer look at the cross-antibiotic (i.e., ceftazidime and cefotaxime) resistance for different subgenic regions of *bla*_CTX-M-14_ revealed that significant enrichment of synergistic pleiotropy was limited to four regions spanning nucleotide positions 82–107, 226–236, 705–716, and 812–825, respectively (highlighted in fig. 3*B*), located in the H1 helix, H2 helix, B3 β-strand, and H11 helix. On the contrary, no subgenic regions were detected with significant antagonistic pleiotropy.

Besides varying responses to different antibiotics, the same mutation may have different effects under different concentrations of the same antibiotic. Indeed, a comparison of relative growth in the presence of different ceftazidime concentrations (fig. 3*C* and *D*; supplementary tables S2, S5, and S6, Supplementary Material online) revealed that, although the effects of most mutations were highly reproducible across concentrations (labeled red dots), some mutations, including a few conferring high-level resistance, were not (labeled gray dots). This finding is in line with the observed nonlinear relationship between bacterial fitness and antibiotic resistance (fig. 3*E*). Such nonlinearity can be understood as another type of pleiotropy, as it essentially means the same mutation confers greater benefit to the bacterium at low antibiotic concentrations, compared with its effect at high antibiotic concentration.

Using our dataset, we can also gauge the existence of epistatic interactions within genes, another factor which has significant effects on evolution (Weinreich et al. 2006; Lehner 2011; Breen et al. 2012). For example, a resistance-increasing mutation might confer decreased resistance when it occurs in a different genetic background if (sign) epistasis is prevalent. To this end, we estimated epistasis within *bla*_CTX-M-14_ from the relative growth of 4849 N2 mutants (i.e., those with two single-nucleotide mutations) and 2165 N1 mutants (i.e., single mutants) (see Materials and Methods). We found that only 38% of the 4849 N2 mutants displayed significant epistasis (supplementary fig. S6, Supplementary Material online). A slight bias for negative epistasis could be found among all N2 mutants (57%, *P* < 10^−20^, binomial test), which further strengthened among those with significant epistasis (65%, *P* < 10^−38^, binomial test). Furthermore, we found that individual single-nucleotide mutations conferring increased ceftazidime resistance tended to be more frequently observed in *bla*_CTX-M-14_ mutants (containing one or more single-nucleotide mutations) with relative growth ≥2 (Spearman's * ρ*  = 0.68, *P* < 10^−5^. fig. 3*F*). This suggests that key resistance-defining mutations contributed to AMR irrespective of other prior mutations, and that epistatic effects within *bla*_CTX-M_ had a minor effect on the evolution of AMR in this context. More importantly, the scarcity of intragenic (sign) epistasis made it more feasible to computationally predict the evolutionary increase in AMR on the basis of relative growth.

### Prospective and Retrospective Prediction of *bla*_CTX-M-14_ Evolution Using Deep-Learning Models

**Fig. 4.**
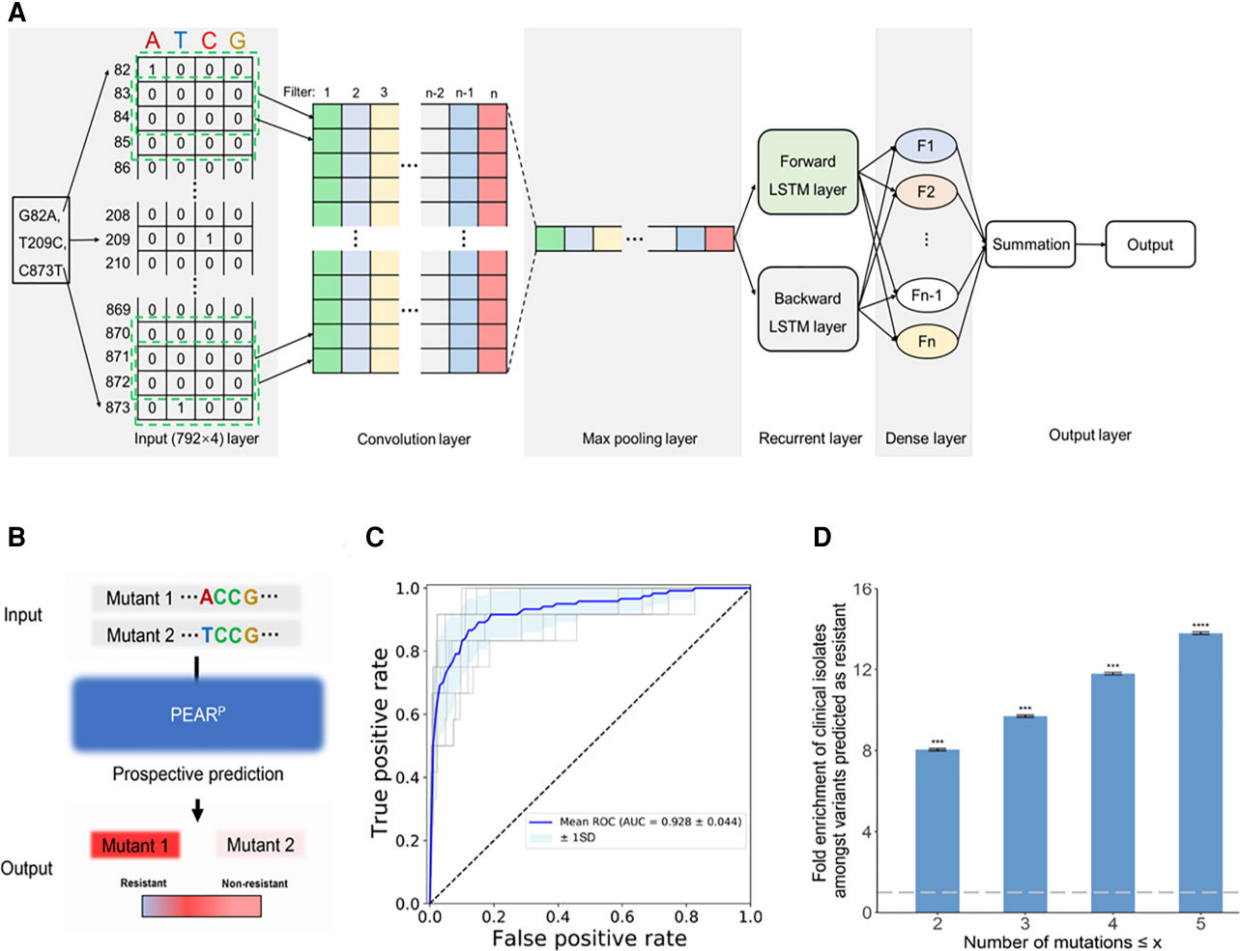
Prospective binary resistance prediction model (PEAR^P^) and its clinical application. (*A*) Graphical illustration of the PEAR model: first, a genotype was transformed to a 792 × 4 matrix for input. The result was passed to a convolution layer and max-pooling layer to extract features and reduce the number of parameters, respectively. A subsequent BLSTM layer considers features from different regions, which are output to a fully connected final layer that summarizes the information learned by the network. (*B*) Schematic illustration of the prospective PEAR^P^ binary classification model. The inputs are mutant DNA sequences, and the output is a prediction of whether the sequence confers resistance to a given antimicrobial. (*C*) Receiver operating characteristic analysis of the test set of phenotypic variants characterized in our experiments. Ten gray lines represent ten different receiver operating characteristic curves from different random splits of the total dataset (80% training set, 10% validation set, and 10% test set). The blue line represents the mean AUC of the 10 gray lines. The light blue area represents the mean ± SD of the AUC. (*D*) Enrichment of clinically isolated mutant alleles among variants predicted to confer resistance by PEAR^P^, relative to all mutants without nonsense mutations. The enrichment (*y* axis) increases as the maximum number of mutations (*x* axis) increases (details in section Materials and Methods). The *P* values of hypergeometric tests with the null distribution of all mutants are indicated. ***P < 10^−6^, ****P < 10^−9^. The error bar represents the standard error. AUC, area under the curve.

The accurate prediction of AMR gene evolution has been challenging but could be highly relevant for the development of clinical management strategies. Our comprehensive fitness landscape with unprecedented coverage could be used to assist in the development of predictive models in two ways. First, the prospective prediction of resistant genotypes is fundamental to prioritizing and tracking of novel mutations that my pose a risk and to designing alternative treatment strategies to minimize their emergence. To this end, we combined convolutional neural networks and bidirectional long short-term memory (BLSTM) networks to create a deep-learning model (fig. 4*A*, see Materials and Methods) referred to as the PEAR model. We implemented PEAR^P^, a binary (AMR increased relative to wild-type *bla*_CTX-M-14_ or not) predictor for novel variants of *bla*_CTX-M-14_ (fig. 4*B*). The PEAR^P^ model was trained on 80% of phenotypic variants characterized in our experiment and achieved an area under the curve (AUC) of 92.8% in a receiver operating characteristic curve (ROC) analysis (fig. 4*C*; see also supplementary fig. S7, Supplementary Material online; and Materials and Methods). Furthermore, we constructed 23 *bla*_CTX-M-14_ mutants not captured by our high-throughput experiment, of which 11 were predicted by PEAR^P^ to conferred increased ceftazidime resistance compared to wild-type *bla*_CTX-M-14_, and 20 of these predictions were confirmed by individual experimental assessment of their MICs (supplementary table S12, Supplementary Material online). More importantly, *bla*_CTX-M-14_ variants with one (N1) to five (N5) single-nucleotide mutations predicted to confer resistance by PEAR^P^ were significantly enriched among *bla*_CTX-M-14_ variants identified in clinical isolates (fig. 4*D*).

**Fig. 5.**
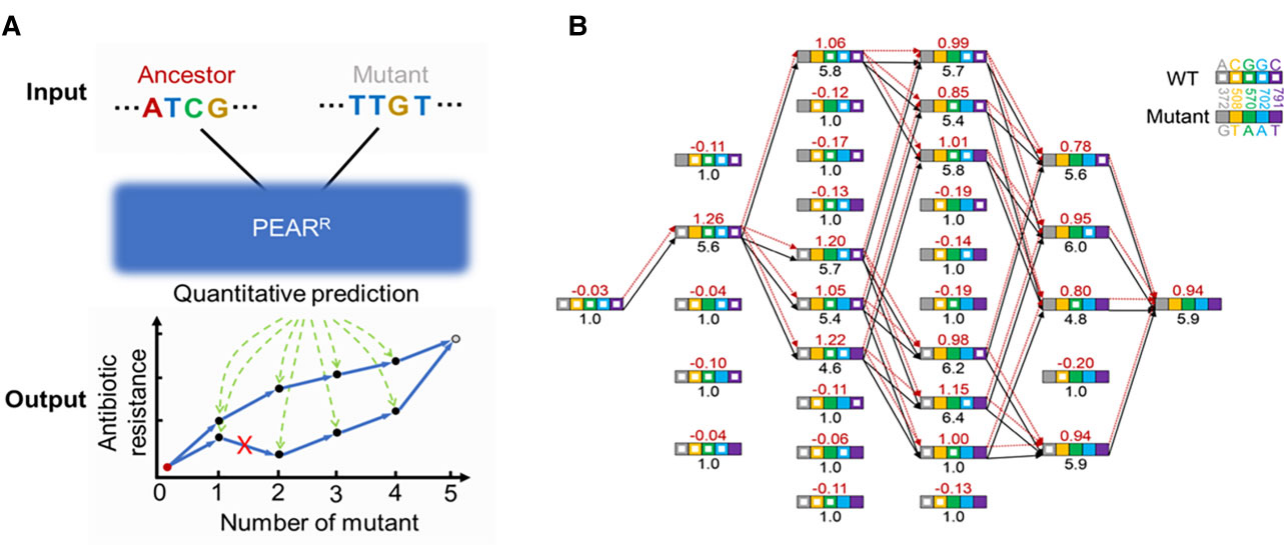
Retrospective prediction of the evolutionary path. (*A*) Schematic illustration of the PEAR^R^ regression model, which is able to model plausible evolutionary trajectories for mutants similar to *bla*_CTX-M-14/14_ based on the comparison of the relative growth of input genotypes. The inputs are ancestral and final genotypes, and the output is a prediction of whether there is a plausible evolutionary trajectory under the assumption that the predicted changes in relative growth for each mutational step should be the best among all alternatives and not detrimental. (*B*) The predicted evolutionary trajectories of a clinical isolate with five single-nucleotide substitutions in the ancestor of *bla*_CTX-M-14_. The black numbers and lines represent log_2_(MIC) values and corresponding evolutionary trajectories (see Materials and Methods), respectively. The red numbers and lines represent the log_10_(relative growth) from predictions and the corresponding evolutionary trajectories, respectively. Filled colored squares reflect corresponding mutations at the position, as shown in the top right legend.

Second, retrospective prediction can be used to identify the evolutionary origin/trajectories of newly emerged AMR gene variants. To accomplish this, we implemented PEAR^R^ (fig. 5*A*), which provides a quantitative score for each genotype, with an intermediate correlation (Pearson's *R* = 0.48, supplementary fig. S8*A*, Supplementary Material online) with the actual level of resistance (MIC). Upon further analysis of the correlation between observed and predicted relative growth rates, PEAR^R^ performed reasonably well for ordinal (but not quantitative) analysis, especially for predicted relative growth for mutants with relative growth ≥2 (supplementary fig. S8*B*, Supplementary Material online). Although this approach was insufficient for predicting MICs directly, it may have the necessary accuracy to predict evolutionary trajectories from ancestral to novel CTX-M variants, which are essential for the origin-tracing of drug resistance. We tested this notion using the PEAR^R^ score on a group of 31 *bla*_CTX-M-14_ mutants, which contain all combinations of mutations found in a clinically isolated *bla*_CTX-M-14_ strain (*bla*_CTX-M-219_, with five mutations from *bla*_CTX-M-14_) (fig. 5*B*). The actual MICs of these 31 *bla*_CTX-M-14_ mutants were also experimentally determined (supplementary table S3, Supplementary Material online). Under the assumption that each mutational step can only move towards the most resistant genotype (or multiple equally resistant genotypes, considering measurement error; see Materials and Methods), we found that the PEAR^R^ score predicted the evolutionary trajectories of *bla*_CTX-M-14_ in a manner compatible with that revealed by actual MICs. Similar findings can be made for CTX-M-4M, a CTX-M-14 variant with four single-nucleotide mutations and dramatically increased relative growth (supplementary fig. S9, table S3, Supplementary Material online). Thus, PEAR^R^ provides an accurate indicator of whether it is possible to evolve from *bla*_CTX-M-14_ to a given variant.

## Discussion

In this study, we measured the fitness landscape of *bla*_CTX-M-14_ variants by evaluating relative growth under selection with varying concentrations of ceftazidime and cefotaxime. The mutations associated with the risk of cross-resistance to both antibiotics were characterized along their nonrandom distribution within the gene. Furthermore, CTX-M-14 mutants that showed a clear growth advantage in our experiment were enriched among clinical isolates, highlighting the potential clinical application of fitness landscape measurements. Additional studies focused on the resistance-associated mutations indicated that their effects were generally unaffected by other mutations, a feature that may facilitate computation of resistant genotypes. Finally, using the measured landscape, we constructed a sequence-based neural network in order to predict the antibiotic resistance of CTX-M-14 mutants. With an AUROC of 92.8%, this predictor can be used for the preliminary assessment of novel CTX-M-14 mutants via the prospective prediction of antibiotic resistance and for the resolution of their origin via the retrospective prediction of their evolutionary trajectory.

There are a few caveats of our study that are worth discussing. First, we only examined single-copy CTX-M-14 variants in a single host strain background, and as a consequence we were unable to explore the implications of intergenic epistasis (i.e., gene–gene interactions) or copy number variation, which may occur in nature (Firnberg et al. 2014). Second, our study focused solely on the effects of antibiotic selection on bacterial fitness in vitro. There is, however, evidence that in vivo AMR evolution can be influenced by additional factors including host immunity and potentially broader interactions with the microbiome (zur Wiesch et al. 2011; Folkesson et al. 2012; Barroso-Batista et al. 2015; Davies et al. 2019). Exploring these interactions would be an interesting future research direction. Third, our experimental design focused on cell autonomous mechanisms of antibiotic resistance. Some AMR genes, such as NDM β-lactamase, encode lipoproteins anchored to the outer membrane in Gram-negative bacteria and this specific cellular localization could lead to the secretion of its variants in outer membrane vesicles (Gonzalez et al. 2016; Lopez et al. 2019). These secreted enzymes may provide a growth advantage for neighboring cells under antibiotic selection. Thus, to measure the fitness of such genes with high precision, deletion of signal peptide to change the cellular localization would be necessary. Fourth, other than plasmid-mediated AMR genes, many of the drug resistance mutation occurs on the essential genes of bacteria, which may hamper the measurement of fitness landscape because genetic manipulation of these genes can be challenging. Developing novel methods for in situ gene editing, such as CRISPR-guided DNA polymerase(Halperin et al. 2018), would be an interesting direction for future research. Fifth, although the observed scarcity of epistasis facilitated the prediction of AMR gene evolution in this study, similar attempts targeting other genes might still be hindered by pervasive epistasis or the dominance of mutations with weak to undetectable effects. In addition, the PEAR^R^ model we constructed appeared sufficient for ordinal analysis, but not for quantitative analysis. Even for ordinal analysis, the application of the PEAR^R^ model might still be limited given that mutants with relative growth <2 tend to have lower Spearman's correlation between biological replicates (supplementary fig. S2D, Supplementary Material online). Therefore, further efforts are apparently needed to improve the accuracy of fitness measurement. Sixth, it has been suggested that availability of different competitors might affect intrinsic cell fitness (Marusyk et al. 2014), thereby altering the fitness measured in different culture stages here. This phenomenon could at least be partially resolved by barcode tracking via sampling with higher temporal densities. Finally, we determined the relative growth of ∼23,000 mutants. However, the investigation of an even larger fitness landscape, including intergenic epistasis that was not covered by our variant library, could likely improve the performance of the prediction model further.

Notwithstanding these limitations, our work brings both technical and conceptual innovations to the field of AMR prediction. Our experimental framework enabled the simultaneous measurement of over 20,000 synthesized *bla*_CTX-M-14_-like variants by overcoming two major technical hurdles. First, the site-specific genomic integration of a single copy of the *bla*_CTX-M-14_ expression cassette ensured an even, identical dosage of a given mutant genotype in each cell, allowing reasonable comparisons among cells/genotypes during competitive growth. Specifically, our experimental pipeline for genomic integration was highly efficient and reproducible. Second, we combined accurate barcode-genotype mapping using long reads (PacBio) with high-throughput barcode frequency measurements using short reads (Illumina HiSeq), allowing a reliable estimation of the functional landscape of CTX-M-14 variants. Our experimental framework and the developed models of resistance prediction will likely be widely applicable for the evaluation of other AMR genes.

We have shown for the first time that subgenic regions within *bla*_CTX-M-14_ are enriched with mutations conferring cross-resistance to both ceftazidime and cefotaxime. Such fine-scale resolutions for pleiotropy are essential for clinical and evolutionary studies focusing on collateral sensitivity/resistance (Rodriguez de Evgrafov et al. 2015; Baym et al. 2016; Imamovic et al. 2018; Nichol et al. 2019; Rosenkilde et al. 2019). Our results also suggested that nonlinear changes in hydrolysis kinetics due to mutations are pervasive. For example, variant G544A, which has a significant growth advantage compared to variant A506G at high antibiotic concentration (1 × MIC), becomes indistinguishable with variant A506G in terms of growth rate at low antibiotic concentration (0.25 × MIC). Traditional therapies involve treating the infected patients with high doses of antibiotics for an extended period of time to eliminate the infecting bacteria. But this practice may lead to an increased risk of antibiotic resistance. Our observations suggest another possible approach in which the most resistant variants can be suppressed by competition with less resistant variants, thereby allowing the administration of prolonged antibiotic therapy (Ardal et al. 2020). In accordance with our theory, multiple studies have shown that adaptive therapy involving repeated, short interval applications of antibiotics can significantly stall disease progression in patients with infectious disease and reduce the risk of the evolution of antibiotic resistance (Ibrahim et al. 2004; Nuttall 2012; Baker et al. 2018), although optimal treatment strategies (antibiotic concentration, dosing timing and frequency, etc.) must be evaluated on a case-by-case basis.

Finally, as demonstrated in this study, computational predictions of the evolution of antibiotic resistance based on the local fitness landscape may have broad clinical applications. They can be used, for instance, in assessing the likelihood that novel variants of a given AMR gene will increase resistance. Novel antibiotics can also be evaluated using these prediction models in order to identify potential resistance alleles that may emerge after their introduction into clinical practice. When applied to evolutionarily intermediate genotypes, this approach could also provide insight into the evolutionary origin of novel resistant genotypes by predicting possible evolutionary trajectories. More importantly, a comprehensive in vitro understanding of the functional landscape of antibiotic resistance, such as that obtained in our study, can facilitate the design of evolutionary control strategies (Nichol et al. 2015; Maltas and Wood 2019; Iram et al. 2021) that could potentially prevent the evolution of antibiotic resistance.

## Materials and Methods

### Construction of the Plasmid Library of *bla*_CTX-M-14_ Variants

The template plasmid was constructed via two rounds of PCR amplification. First, the *bla*_CTX-M-14_ gene with its native promoter was amplified from genomic DNA extracted from clinical isolates (Tian et al. 2016), and the *rrnB* terminator was amplified from the integrated pOSIP-KH plasmid. After the purification of the PCR product, the two DNA fragments were concatenated by fusion PCR to obtain the full expression cassette of *bla*_CTX-M-14_. The expression cassette was subsequently cloned into pMD19-T (Takara, Japan), a high-efficiency cloning vector. Positive colonies were confirmed by PCR and Sanger sequencing. The resulting plasmid was named pMD19-CTX-M-Ter and stored at −20 °C.

The gene variant library was constructed via another two-step PCR approach; the manufacturers’ instructions were followed for commercial kits unless otherwise specified. Phanta Max Super-Fidelity DNA Polymerase (Vazyme, China) was used in all amplification reactions. Doped oligonucleotides were synthesized by IDT (https://www.idtdna.com/). Because the length of chemically synthesized oligonucleotides with degenerate sites containing manually defined nucleotide fractions was limited to 90 nt and invariant regions at both ends of the oligonucleotides were required for PCR, we designed only 50 variable sites for each oligonucleotide, and the leading and trailing 20 nt were invariant sequences identical to those of wild-type *bla*_CTX-M-14_. In addition, for the doped oligonucleotides, each position contained the wild-type nucleotide at a 97% frequency and a 1%:1%:1% mix of the other three nucleotides. As a result, the library exhibited a mutation rate of approximately 3% per nucleotide. The PCR products were used as a template for fusion PCR to add 20 nt random barcodes and restriction sites to each variant (primers: CTX[NP]-SOE-5F-BamHI and CTX-Ter-barcode 3R-PstI; supplementary table S7, Supplementary Material online). After 30 cycles, the PCR products were analyzed by gel electrophoresis, and the target bands were extracted. Purified DNA was digested with FastDigest *BamH*I and FastDigest *Pst*I (Thermo Scientific, America) for 1 h at 37 °C. The digested inserts were purified with the Cycle Pure Kit (OMEGA, USA). In parallel, the plasmid backbone was prepared by digesting pOSIP-KH with the same enzymes at 37 °C for 1 h. The ligation reactions were prepared by mixing 68 ng of insert, 110 ng of digested plasmid, and 1 μl of T4 DNA ligase (Thermo Scientific, USA), followed by incubation for 4 h at 16 °C. The ligation products were transformed into *E. coli* EC100D^TM^  *pir*^+^ using a heat-shock method. To allow plasmid propagation without integration, the Luria broth (LB) agar plate was incubated at 30 °C for 20 h. Final library diversity was estimated to be approximately 200,000 clones. All colonies were collected from LB agar plates by washing with LB liquid medium. Pooled plasmid variants were extracted with a Plasmid Midi Kit (OMEGA, USA) and stored at −80 °C.

### Site-Directed Mutagenesis

For 543 single-nucleotide *bla*_CTX-M-14_ mutations that were not captured by PacBio CCS, we constructed the corresponding mutants via site-directed mutagenesis (primers in supplementary table S8, Supplementary Material online), in which a two-step PCR procedure was carried out to replace the indicated site. Specifically, two simultaneous amplification reactions were performed, and both PCR products were gel purified using a gel extraction kit (OMEGA, USA). To obtain full-length mutated fragments with corresponding barcodes, purified DNA from the first round of PCR was mixed in equimolar concentrations and used as a template for the second round of PCR. After purification by gel electrophoresis, the full-length DNA fragment was digested using FastDigest *BamH*I and FastDigest *Pst*I. The digested fragment was cloned into pOSIP-KH. The ligation product was transformed into *E. coli* EC100D^TM^  *pir*^+^ competent cells. Recombinant plasmids were purified, and *bla*_CTX-M-14_ was sequenced to confirm any mutations present. EC100D^TM^  *pir*^+^ chemically competent cells were prepared through a modified Hanahan method (Green and Sambrook 2018). Briefly, a single colony from a fresh plate of the strain was inoculated into 2 ml LB medium and cultivated at 37 °C at 300 rpm overnight as a seed culture. One milliliter of the seed culture was transformed into 100 ml of LB liquid medium and cultivated at 37 °C until the OD_600_ reached a value of 0.4–0.6. Each 25 ml culture was transferred to a chilled 50 ml centrifuge tube and incubated on ice for 15 min. The cell pellet was spun down at 4 °C (4,000 rpm for 10 min), and the supernatant was discarded. The cell pellet was then resuspended in 30 ml of ice-cold 0.1 M CaCl_2_-MgCl_2_ 280 mmol/l MgCl_2_ and 20 mmol/l CaCl_2_ solution, followed by incubation on ice for 30 min. After being spun down again 4,000 rpm at 4 °C for 5 min, the cell pellet was resuspended in 2 ml of iced 0.1 M CaCl_2_–15% glycerol, and 100 μl aliquots of the suspension were transferred to 1.5 ml microtubes and stored at −80 °C.

To assess the accuracy of the measured relative growth, 54 variants of *bla*_CTX-M-14_ were also constructed by one-step cloning. Briefly, two adjacent mutated target fragments with 20–15 bp homologous sequences were amplified by PCR. The PCR products were gel purified using a gel extraction kit (OMEGA). The pOSIP-KH plasmid was digested with FastDigest *BamH*I and FastDigest *Pst*I at 37 °C for 1 h. Ligation was performed at a vector:insert ratio of 1 : 3 using a Seamless Cloning Kit (Beyotime, China). The product was transformed into *E. coli* EC100D^TM^  *pir*^+^ competent cells and selected on kanamycin (25 μg/ml). Recombinant plasmids were purified, and the corresponding *bla*_CTX-M-14_ variant was sequenced to confirm the mutation.

### Construction of the *E. coli* MG1655 Strain Pool

To construct the *bla*_CTX-M-14_ variant strain pool, the plasmid library was transformed into *E. coli* MG1655 by electroporation. Immediately after electroporation, 1 ml SOC medium was added, and the culture was recovered at 37 °C for 1 h and then plated on an LB agar plate containing 25 μg/ml kanamycin. Subsequently, LB agar plates were incubated at 37 °C for 12 h. Over 300,000 colonies were collected from LB agar plates by washing with LB liquid medium.

### Competition Experiments

After harvesting the *E. coli* MG1655 strain pool, 100 μl aliquots (∼2 × 10^9^ CFU) of the strain pool were added to 500 ml of LB liquid medium containing 1 μg/ml (0.25 × MIC), 2 μg/ml (0.5 × MIC), or 4 μg/ml (1 × MIC) ceftazidime or 128 μg/ml (1 × MIC) cefotaxime (supplementary table S9, Supplementary Material online). Three replicate competition experiments were performed. To dynamically examine the change in the phenotype, 50 ml of each sample was collected at the indicated culture stage (when the OD_600_ was 0.23, 0.4, or 0.7). At each culture stage, bacterial cells were diluted tenfold and plated on LB agar media. CFUs were counted after 14 h of incubation (supplementary fig. S10, Supplementary Material online).

### Library Preparation

For PacBio sequencing, the plasmid library was used as the template, and 25 cycles of PCR were performed to amplify the expression cassette, including the barcode. The PCR product was run on an agarose gel and purified with a gel extraction kit (OMEGA).

For HiSeq sequencing, genomic DNA was extracted from the sample of interest. Two rounds of PCR were performed to amplify the barcode from the *E. coli* MG1655 genome. In brief, 20 cycles of PCR were performed to amplify the barcode-containing fragment, and the purified product was used as the template for the second round of PCR. Twenty-five additional cycles of PCR were then performed to amplify the barcode sequence using primers that also added Illumina TruSeq adapters.

### Flow Cytometry Analysis

*E. coli* MG1655 electrocompetent cells were transformed with plasmids containing the fluorescent markers pACY-sfGFP (p15A ori, CmR) and pACT-mCherry (p15A ori, CmR). To ensure that the expression of the fluorescent protein did not affect the experimental outcome, a validation experiment was performed in which the growth of sfGFP-labeled *E. coli* MG1655 versus that of mCherry-labeled *E. coli* MG1655 was evaluated in the absence of ceftazidime. Before each experiment, sfGFP-labeled *E. coli* MG1655 (pOSIP-KH-CTX-M-14_WT_) and mCherry-labeled *E. coli* MG1655 (pOSIP-KH-CTX-M-14_mutant_) were grown separately until they reached the exponential growth phase in each population. Then, the cell cultures were mixed at a 1 : 1 ratio and grown in the presence of ceftazidime. The ratio of labeled cells was confirmed by flow cytometry in the initial culture. The mixed population in the log phase of growth was analyzed by flow cytometry to immediately assess the ratio between mCherry-positive and sfGFP-positive cells. The competitive index is calculated as (*M*_t_/*M*_0_)/(WT_t_/WT_0_), whereas *M*_t_ and *M*_0_ are, respectively, the frequency of mCherry-labeled mutant cells at the beginning and end of the co-culture, and WT_t_ and WT_0_ are, respectively, the frequency of sfGFP-labeled WT cells at the beginning and end of the co-culture. Results of this experiment were shown in fig. 3*E*.

### Antimicrobial Susceptibility Testing

The MICs of cefotaxime and ceftazidime for *E. coli* MG1655 carrying the *bla*_CTX-M-14_ or *bla*_CTX-M-14_ mutant were determined using the agar dilution method and interpreted using breakpoints defined by the Clinical and Laboratory Standards Institute.

### Associating Barcodes and *bla*_CTX-M-14_ Genotypes via PacBio Sequencing

We used three single-molecule real-time cells on the PacBio Sequel platform to sequence the constructed plasmid pool and obtained a total of 3.5 × 10^7^ raw subreads (supplementary fig. S1*A*, Supplementary Material online). There was a non-negligible probability of the presence of heterologous dsDNA molecules since the ssDNA molecules of different mutants in the plasmid pool were highly similar and therefore capable of forming heterologous duplexes. To avoid base-calling errors caused by heterologous dsDNA, we used BLASR with default parameters (Chaisson and Tesler 2012) to map all subreads of each zero-mode waveguide to the wild-type sequence of *bla*_CTX-M-14_ and divided them into positive and negative strands. We then used the CCS algorithm (*–min-length 900 –max-length 1600 –min-passes 5*) (Wenger et al. 2019) to call consensus sequences separately from the subreads derived from positive and negative strands, with at least five subreads each, which corresponded to a base-calling error rate ≤1% (supplementary fig. S1*B*, Supplementary Material online). From each CCS without any indels, we extracted the associated barcode-genotype pair. Due to the occurrence of template switching events during PCR amplification, one barcode might be assigned multiple genotypes to some extent. To improve the quality of barcode matching to specific genotypes, we applied a maximal parsimonious strategy to accept only one association supported by most PacBio reads or discarded the barcode if this strategy failed.

To verify the accuracy of the PacBio-derived association between a given mutant genotype and barcode, we randomly picked 14 transformant colonies and used Sanger sequencing to determine the barcodes and the associated mutant *bla*_CTX-M-14-like_ genotype. Among the assigned barcode-genotype pairs, 13/14 were in complete agreement with the Sanger sequencing results; the remaining mismatched barcode-genotype pair was compatible with a template switch during PCR.

### HiSeq Sequencing and Relative Growth Estimation

We performed paired-end 150 bp sequencing on each sample on the Illumina HiSeq XTen platform, with an estimated sequencing depth of 100, to obtain the frequency of each barcode within a sample. Barcode sequences of 20 nt were extracted from the sequencing reads, and the barcodes with nonidentical sequences from matching forward and reverse sequencing reads were excluded from further analysis. In addition, barcodes captured by PacBio technology were included in the downstream analysis. Barcode counts across technical repeats or biological replicates were combined (supplementary table S10, Supplementary Material online). To ensure accurate estimation of the relative growth, 25,520 genotypes with a total of at least 100 reads from the three technical repeats at CS0 were analyzed.

Relative growth at each culture stage of each competitive growth assay was calculated for individual mutations as [*f*_*t*_(Mutant)/*f*_0_(Mutant)]/[*f*_*t*_(WT)/*f*_0_(WT)], where *f_t_* is the frequency of the barcode of a certain genotype in a postcompetition sample; *f*_0_ is the frequency of the barcode of the same genotype at CS0; *f*_*t*_(WT) is the frequency of the wild-type barcode in a postcompetition sample; and *f*_0_(WT) is the frequency of the wild-type barcode at CS0 (supplementary fig. S10, Supplementary Material online).

To estimate the reliability of the relative growth measurements, we used genotypes with at least three barcodes to calculate the SNR. We calculated the observed standard deviation (*SD*) of the *f*_*t*_/*f*_0_ for different barcodes of each genotype. We then randomly perturbed the correspondence between genotype and barcode and recalculated the perturbed SD (*SD*′) of the *f*_*t*_/*f*_0_ for different barcodes of each genotype. We repeated the perturbance 1,000 times to obtain 1,000 *SD*′ values, whose average value was represented as $\bar{SD^{\prime }}$. The SNR was then calculated as $\mathrm{SNR}=\frac{1}{n}\sum_{i\;=\;1}^{n}\frac{\bar{S{D^{\prime }}_{i}}}{SD_{i}},$ where *n* is the number of genotypes with at least three barcodes.

### Analysis of Three-Dimensional Structure

Three-dimensional structure data with the highest available resolution (Protein Data Bank [PDB]: 6D7H-CTX-M-14 apoenzyme) were downloaded from the PDB database. To identify the enriched regions of the antibiotic resistance mutants, we calculated the mean log_10_(relative growth) value for all amino acids within 10 Å interval for each position. The relative growth of each amino acid is the maximum relative growth of focal-codon single-nucleotide mutants. The distance between two amino acids is represented by the distance between their alpha-carbon atoms in three-dimensional space, as extracted by PyMOL 2.3.2.

### Calculating the CAI

The coding sequence of MG1655 (https://www.ncbi.nlm.nih.gov/nuccore/NC_000913) was downloaded to calculate the CAI according to the following formula:

$$w_{i}=\frac{\;f_{i}}{\max(\;f_{j})}$$

 

$$\mathrm{CAI}={\left(\overset{L}{\underset{i=1}{\prod }}\;w_{i}\right)}^{\frac{1}{L}}$$

For each amino acid, *w_i_* represents the weight of each of its codons as the ratio between frequency of the codon *f_i_* and frequency of the codon of maximal frequent synonymous codon *f_j_* for that amino acid. *L* represents the number of codons of a variant, and the CAI is defined as the geometric mean of the weight associated to each codon over the length (*L*).

### Estimating Epistasis from Relative Growth Values

Epistasis is determined as ɛ = *r*_*AB*_ − *r*_*A*_*r*_*B*_, where *r_AB_* is the relative growth of a N2 mutant and *r_A_*and *r_B_* are the fitness of the two corresponding N1 mutants. To examine if ɛ varies significantly from 0, we assessed from each of the three biological replicates and conducted a *t* test.

### Neural Network Construction and Evolutionary Trajectory Prediction

To estimate the complicated genotype–phenotype relationships, we constructed two neutral networks using the Keras module of TensorFlow 2.2.0. The first neutral network is a binary classification model named PEAR^P^, which was used to classify a given genotype as resistant or not. According to the relative growth values in 0.5 × MIC ceftazidime (supplementary tables S5 and S11, Supplementary Material online), we divided the genotypes according to two classification labels: nonresistant mutants (log_10_(relative growth) < 1) and resistant mutants (log_10_(relative growth) ≥ 1). We split our dataset into a training set, a validation set, and a test set (80%, 10%, and 10% of the total data by stratified random sampling, respectively) and used binary-cross-entropy as the loss function to predict labels from the observed genotypes. This model included six main layers: the input, convolution, max pooling, recurrent, dense, and output layers. The input layer consisted of a 792×4 one-hot encoded matrix of genotypes, in which mutants were recorded as 1 and wild-type genotypes as 0. The convolution layer with rectified linear unit activation (ReLU) extracted sequence-feature information using 32 filters of 5 kernel sizes. The max-pooling layer included 13 pool sizes and 13 strides to reduce computational complexity and prevent overfitting. The subsequent BLSTM network layer could process sequences in both the forward and backward directions using two separate LSTMs, improving the recognition of features in different regions and integrating them. The dense layer (also consisting of rectified linear units) was then used to integrate information from the BLSTM networks. The final layer transforms the classification probability using a sigmoid nonlinear function. Since the number of nonbeneficial and beneficial genotypes (nonbeneficial: beneficial = 23660 : 123) was highly imbalanced, we adopted upsampling and higher weighting to correct the training set's imbalance. A drop-out layer was also added to prevent overfitting. All optimizations were performed using five replicates from several random initial weights, and the results showing the lowest loss (loss function = binary-cross-entropy) for the validation set were chosen.

We randomly repeated the procedure ten times to split the total dataset (80% training set, 10% validation set, and 10% test set) to build different PEAR^P^ models. Then, the PEAR^P^ model exhibiting the highest AUC with the test set was used to predict whether a genotype was resistant. All mutants containing one single-nucleotide mutation (N1 = 2308) or two single-nucleotide mutations (N2 = 2657862) or a randomly selected subset of genotypes with three (N3 = 3000000), four (N4 = 4000000), and five (N5 = 5000000) single-nucleotide mutations (excluding nonsense mutations) as well as clinical isolates with 1–5 single-nucleotide mutations were included as the subjects for prediction. We considered the mutants with a resistance probability ≥ 0.955, such that the false positive rate was minimized while the true positive rate remained above 0.5.

The second neutral network was a regression model referred to as PEAR^R^, which was used to predict the log_10_(relative growth) of a given genotype. We randomly split our relative growth dataset obtained in the presence of 0.5 × MIC ceftazidime into training, validation and testing sets (80, 10, and 10% of total data, respectively) and used the mean-square error (MSE) as the loss function. The basic structure of PEAR^R^ was otherwise the same as that of PEAR^P^, but these two models are in fact different since PEAR^R^ is a regression model; specifically, 1) the convolution layer with leaky rectified linear units (leaky ReLUs) contained 32 filters of 21 kernel sizes; 2) the max-pooling layer included three pool sizes and three strides; and 3) all optimizations were also performed with five replicates from several random initial weights and the results with the best MSE for the test set were chosen.

To trace the possible evolutionary trajectories, we assumed that for each mutational step, only the most resistant genotype(s) will be evolutionarily fixed. The logic behind this assumption is that the most resistant genotype wins the competition with other mutational candidates. Nevertheless, given the measurement error, we might not be able to pin-point exactly the one genotype that is the most resistant. We therefore considered, among the mutational candidates for one particular mutational step, that any genotype whose resistance metric [log_2_(MIC) or log_10_(relative growth)] was within one SD of the highest value as equally resistant. On the basis of this assumption, we aimed to test the performance of PEAR^R^ using a clinical isolate with CTX-M-14 variant with multiple mutations (mutation ≥ 4). To this end, we found four clinical variants of CTX-M-14 with five mutations, including CTX-M-16, CTX-M-51, CTX-M-214, and CTX-M-219. The ceftazidime MICs of these four mutants were experimentally determined (supplementary table S3, Supplementary Material online) and we found that *E. coli* MG1655 carrying CTX-M-51, CTX-M-214, and wild-type CTX-M-14 had similar MICs. *E. coli* with CTX-M-16 exhibited a 4-fold increase in ceftazidime MICs, and notably, the CTX-M-219-producing strain showed the highest MIC of ceftazidime, that is, a 32-fold increase relative to wild-type CTX-M-14. In addition, CTX-M-219 is a novel variant of the CTX-M-14 group. Therefore, CTX-M-219 was used to validate the performance of the model. Next, 31 CTX-M-14 mutants of CTX-M-219 containing all possible combinations of mutations were generated. The MICs of these 31 CTX-M-14 mutants were also experimentally determined. The log2 (MIC) or log10 (relative growth) SD was calculated for 31 genotypes meeting these criteria, as shown in fig. 5*B*. Additionally, CTX-M-4M, a CTX-M-14 variant with four single-nucleotide mutations, which dramatically increased relative growth in our library, was used to further validate the performance of the PEAR^R^ model. Similarly, the evolutionary trajectories of CTX-M-4M matched with PEAR^R^ score (supplementary table S3 and fig. S9, Supplementary Material online).

## Supplementary Material

Supplementary data are available at *Molecular Biology and Evolution* online.

## Supplementary Material

msac086_Supplementary_Data

## Data Availability

Raw sequencing data were deposited in the Sequence Read Archive (SRA) under BioProject number PRJNA687219 (https://dataview.ncbi.nlm.nih.gov/object/PRJNA687219? reviewer=v0blut5uag1lscnhcl7uih8ff8). Processed data sets are available at supplementary table. Custom R and python codes were used in data analysis, which are available on Github (https://github.com/woson2020/CTXM-14).
