# Supplementary material for: Prediction of Antibiotic Resistance Evolution by Growth Measurement of All Proximal Mutants of Beta-Lactamase
Source: Mol Biol Evol. 2022 Apr 29;39(5):msac086. doi: 10.1093/molbev/msac086 (PMC9087888; doi:10.1093/molbev/msac086)
Supplement: msac086_Supplementary_Data [file msac086_supplementary_data.zip › Table_S12_PEARP_model_validation.docx]

**Table S12 PEAR^P^ model validation by additional *bla*_CTX-M-14_ mutants not captured by the high throughput experiment**

| Genotype  (nucleotide) | Genotype  (amino acid) | PEAR^P^ prediction  (Increased ceftazidime resistance relative to wildtype *bla*_CTX-M-14_) | MIC of ceftazidime  (μg/ml)* |
| --- | --- | --- | --- |
| A217G T394C T587A | S73G Y132H L196H | No | 2 |
| A104G T158C C324T | Q35R I53T | No | 8 |
| G87T A331T A545T | Q29H I111F D182V | No | 2 |
| C192T T231C T683C | L228S | No | 2 |
| T162A A320T T394A | D54E N107I Y132N | No | 2 |
| T56A T327A | N109K | No | 2 |
| A372G G570A C791T | T264I | No | 2 |
| G570A G702A C791T | T264I | No | 2 |
| A725G T374C | L125P D242G | No | 14 |
| T281C A497G T796G T797G | L94P D166G F266G | No | 2 |
| A403T C557T | N135Y P186L | No | 2 |
| G544A A253G A275T | K85E Q92L D182N | No | 14 |
| A172G T515G | N58D L172R | Yes | 18 |
| C378T G486A T515G | L172R | Yes | 10 |
| A372G C508T G570A G702A | P170S | Yes | 32 |
| C508T G570A G702A C791T | P170S T264I | Yes | 44 |
| G544A C451A | D182N | Yes | 14 |
| A506G C309G | E169G | Yes | 14 |
| C509G C656T T768A | P170R T219I | Yes | 14 |
| A506G C477G A634T G862C | E169G T212S A288P | Yes | 14 |
| C509G A733T | P170R T245S | Yes | 14 |
| G544A A269T | Q90L D182N | Yes | 14 |
| A725G G330C A340C | K114Q D242G | Yes | 14 |

*: The MIC of wildtype *bla*_CTX-M-14_ is 2 μg/ml
