# Supplementary material for: Prediction of Antibiotic Resistance Evolution by Growth Measurement of All Proximal Mutants of Beta-Lactamase
Source: Mol Biol Evol. 2022 Apr 29;39(5):msac086. doi: 10.1093/molbev/msac086 (PMC9087888; doi:10.1093/molbev/msac086)
Supplement: msac086_Supplementary_Data [file msac086_supplementary_data.zip › Table_S3_MIC_value_of_ceftazidime_for_MG1655_carrying_pOSIP-CTX-M-14_variants.docx]

**Table S3 MIC value of ceftazidime for MG1655 carrying pOSIP-CTX-M-14 variants**

| Mutants | MIC | | |
| --- | --- | --- | --- |
|  | Replicate1 | Replicate 2 | Replicate 3 |
| Wild type CTX-M-14 | 2 | 2 | 4 |
| D179Y (G544A) | 16 | 16 | 16 |
| D240G (A725G) | 16 | 16 | 16 |
| E166G (A506G) | 16 | 16 | 16 |
| L169R (T515G) | 32 | 16 | 32 |
| P167R (C509G) | 16 | 16 | 32 |
| P167S | 64 | 64 | 64 |
| P167L | 16 | 16 | 32 |
| P167H | 32 | 32 | 32 |
| P167A | 32 | 32 | 32 |
| P167T | 32 | 32 | 32 |
| P167C | 4 | 4 | 4 |
| P167D | 32 | 32 | 32 |
| P167E | 16 | 16 | 32 |
| P167F | 16 | 16 | 32 |
| P167G | 64 | 64 | 64 |
| P167I | 16 | 16 | 16 |
| P167K | 32 | 32 | 64 |
| P167M | 32 | 32 | 32 |
| P167N | 32 | 32 | 32 |
| P167Q | 32 | 64 | 32 |
| P167V | 16 | 16 | 16 |
| P167W | 16 | 16 | 16 |
| P167Y | 32 | 16 | 32 |
| CTX-M-219  (A372G C508T G570A G702A C791T) | 40 | 44 | 60 |
| A372G C508T G570A G702A | 32 | 32 | 28 |
| A372G C508T G570A C791T | 44 | 36 | 44 |
| A372G C508T G702A C791T | 24 | 28 | 28 |
| A372G G570A G702A C791T | 4 | 2 | 4 |
| C508T G570A G702A C791T | 44 | 40 | 44 |
| A372G C508T G570A | 40 | 40 | 44 |
| A372G C508T G702A | 40 | 36 | 44 |
| A372G C508T C791T | 44 | 40 | 52 |
| A372G G570A G702A | 2 | 2 | 4 |
| A372G G570A C791T | 2 | 2 | 4 |
| A372G G702A C791T | 2 | 2 | 2 |
| C508T G570A G702A | 36 | 28 | 36 |
| C508T G570A C791T | 44 | 44 | 60 |
| C508T G702A C791T | 24 | 28 | 28 |
| G570A G702A C791T | 2 | 4 | 2 |
| A372G C508T | 40 | 44 | 44 |
| A372G G570A | 2 | 2 | 4 |
| A372G G702A | 2 | 2 | 2 |
| A372G C791T | 2 | 2 | 4 |
| C508T G570A | 40 | 40 | 48 |
| C508T G702A | 40 | 36 | 36 |
| C508T C791T | 24 | 20 | 24 |
| G570A G702A | 2 | 2 | 2 |
| G570A C791T | 2 | 2 | 4 |
| G702A C791T | 2 | 2 | 2 |
| A372G | 2 | 2 | 4 |
| C508T | 36 | 36 | 44 |
| G570A | 2 | 2 | 4 |
| G702A | 2 | 2 | 2 |
| C791T | 2 | 2 | 2 |
| CTX-M-4M (G591A T691G C723G A725G) | 12 | 12 | 12 |
| G591A T691G C723G | 2 | 2 | 2 |
| G591A T691G A725G | 10 | 10 | 10 |
| G591A C723G A725G | 14 | 14 | 14 |
| T691G C723G A725G | 10 | 10 | 10 |
| G591A T691G | 2 | 2 | 2 |
| G591A C723G | 2 | 2 | 2 |
| G591A A725G | 12 | 12 | 10 |
| T691G C723G | 2 | 2 | 2 |
| T691G A725G | 12 | 12 | 12 |
| C723G A725G | 12 | 12 | 12 |
| G591A | 2 | 2 | 2 |
| T691G | 2 | 2 | 2 |
| C723G | 2 | 2 | 2 |
| A725G | 10 | 10 | 10 |
| CTX-M-16 | 8 | 16 | 8 |
| CTX-M-51 | 2 | 4 | 2 |
| CTX-M-214 | 4 | 2 | 2 |
