# Supplementary material for: Prediction of Antibiotic Resistance Evolution by Growth Measurement of All Proximal Mutants of Beta-Lactamase
Source: Mol Biol Evol. 2022 Apr 29;39(5):msac086. doi: 10.1093/molbev/msac086 (PMC9087888; doi:10.1093/molbev/msac086)
Supplement: msac086_Supplementary_Data [file msac086_supplementary_data.zip › Table_S9_MIC_value_of_antibiotics_for_MG1655,_MG1655_carrying_pOSIP-CTX-M-14_and_clinical_isolate.docx]

**Table S9 MIC value of antibiotics for MG1655, MG1655 carrying pOSIP-CTX-M-14 and clinical isolate**

| Antibiotic | MIC (μg/ml) | | |
| --- | --- | --- | --- |
|  | MG1655 | MG1655 carrying pOSIP-CTX-M-14 | clinical isolate |
| Cefotaxime | 0.0625 | 128 | >512 |
| Ceftazidime | 2 | 4 | 64 |
