# Supplementary figures and images for: Prediction of Antibiotic Resistance Evolution by Growth Measurement of All Proximal Mutants of Beta-Lactamase
Source: Mol Biol Evol. 2022 Apr 29;39(5):msac086. doi: 10.1093/molbev/msac086 (PMC9087888; doi:10.1093/molbev/msac086)

**A**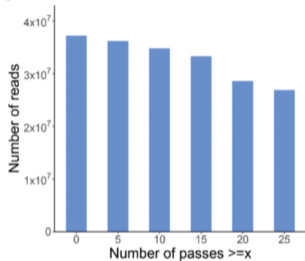**B**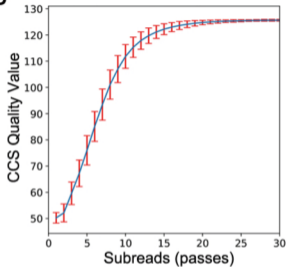

Supplement: msac086_Supplementary_Data [file msac086_supplementary_data.zip › Figure_S1.pdf]

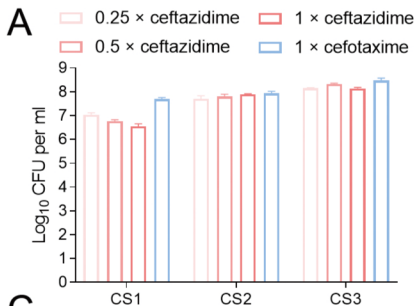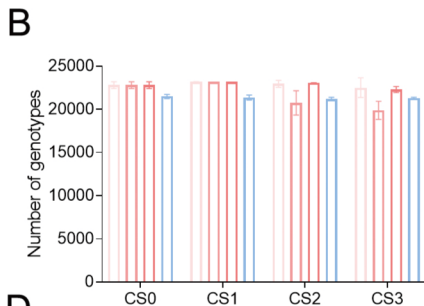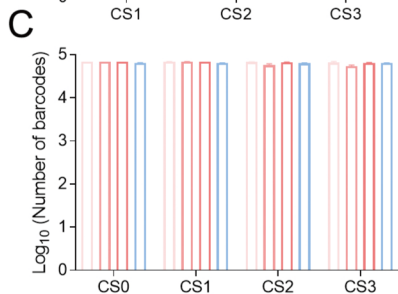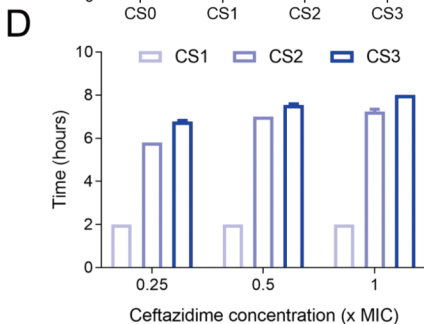

Supplement: msac086_Supplementary_Data [file msac086_supplementary_data.zip › Figure_S10.pdf]

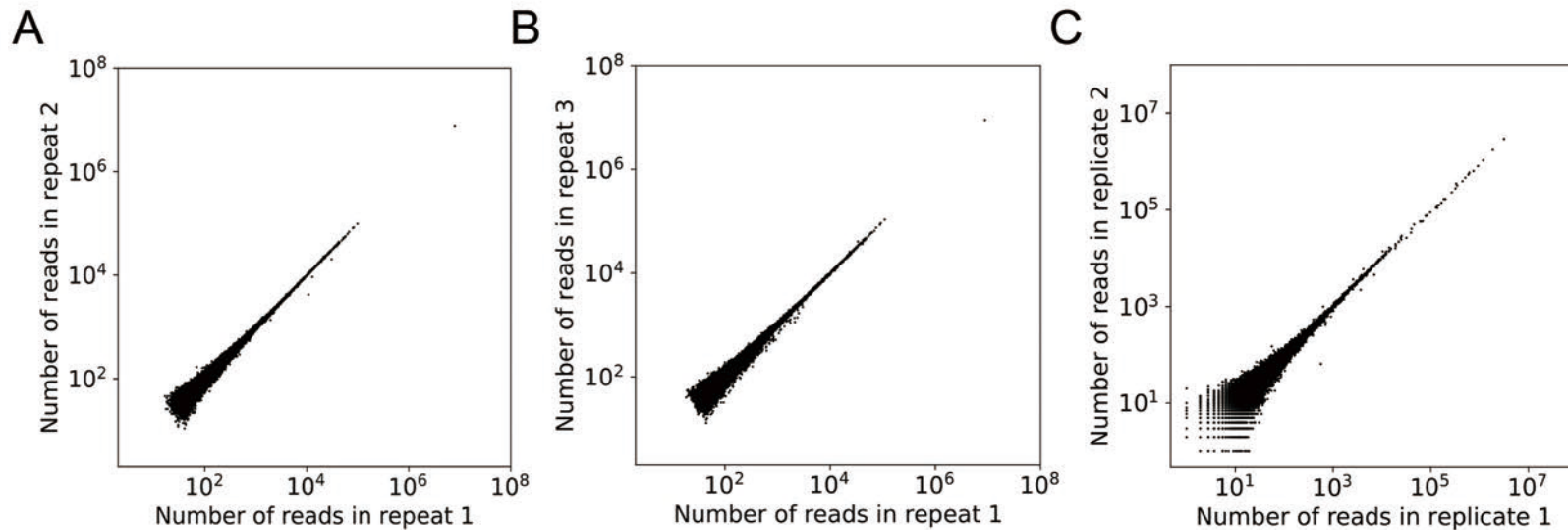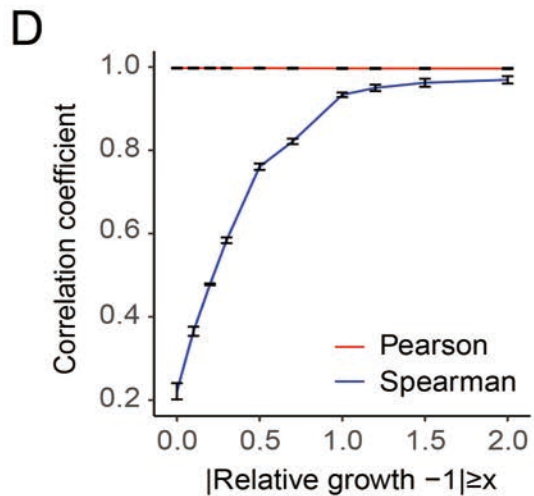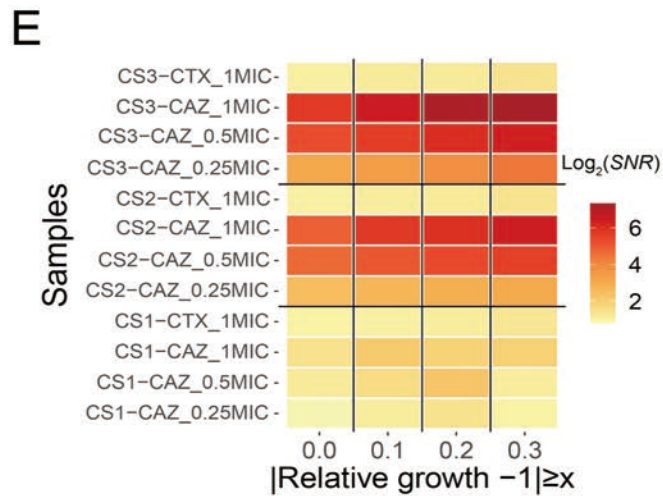

Supplement: msac086_Supplementary_Data [file msac086_supplementary_data.zip › Figure_S2.pdf]

**A**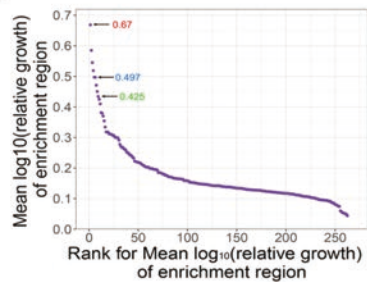**B**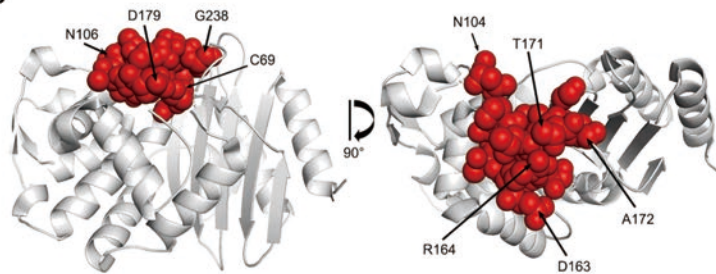**C**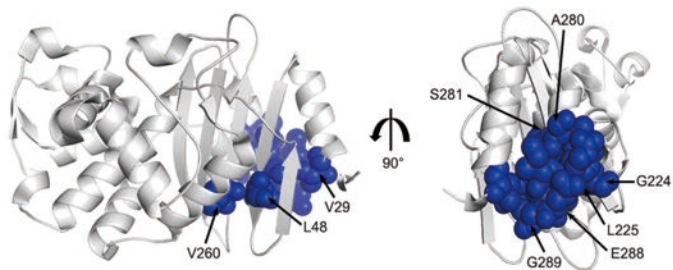**D**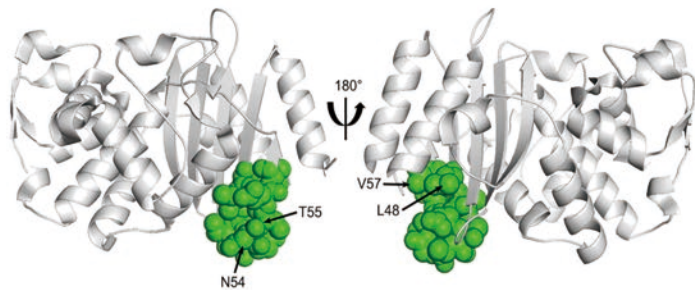

Supplement: msac086_Supplementary_Data [file msac086_supplementary_data.zip › Figure_S3.pdf]

**A**

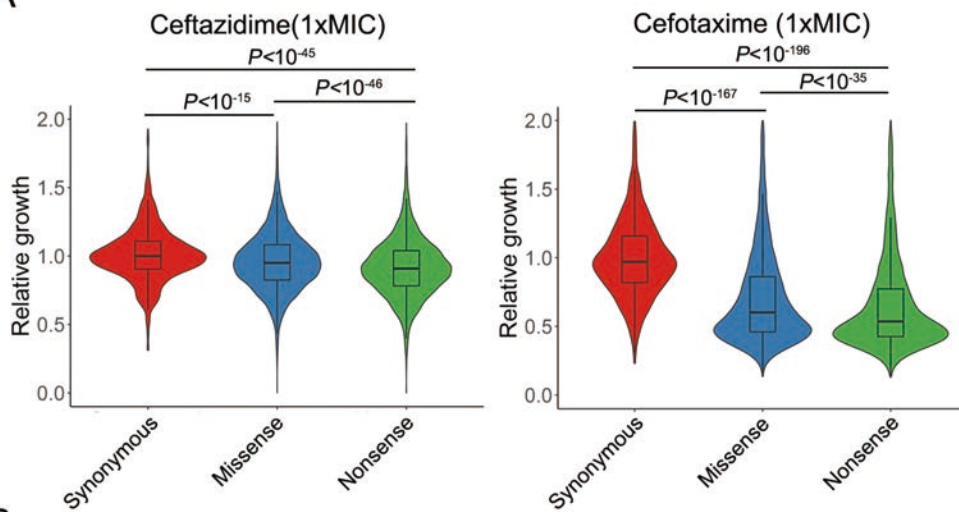

**B**

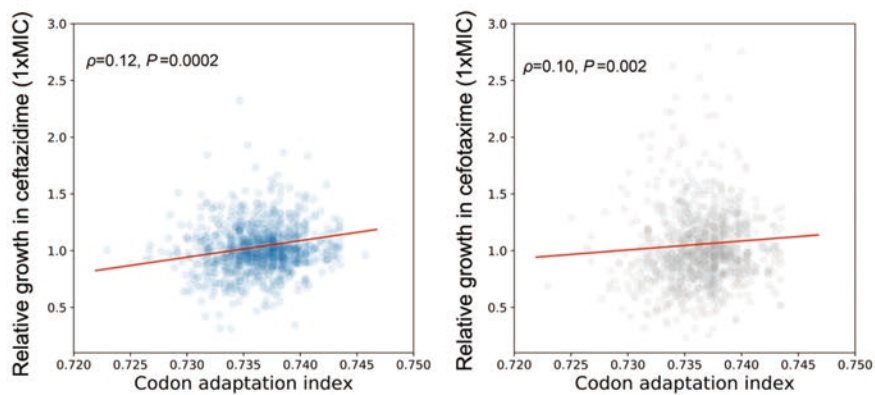

Supplement: msac086_Supplementary_Data [file msac086_supplementary_data.zip › Figure_S4.pdf]

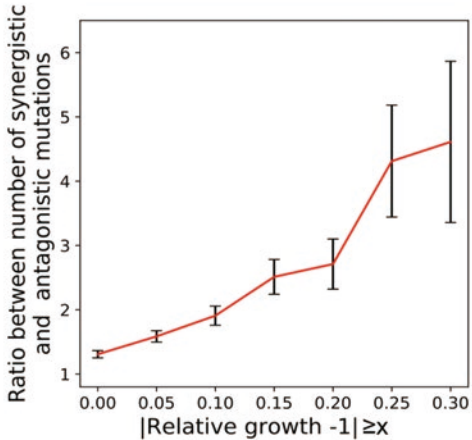

Supplement: msac086_Supplementary_Data [file msac086_supplementary_data.zip › Figure_S5.pdf]

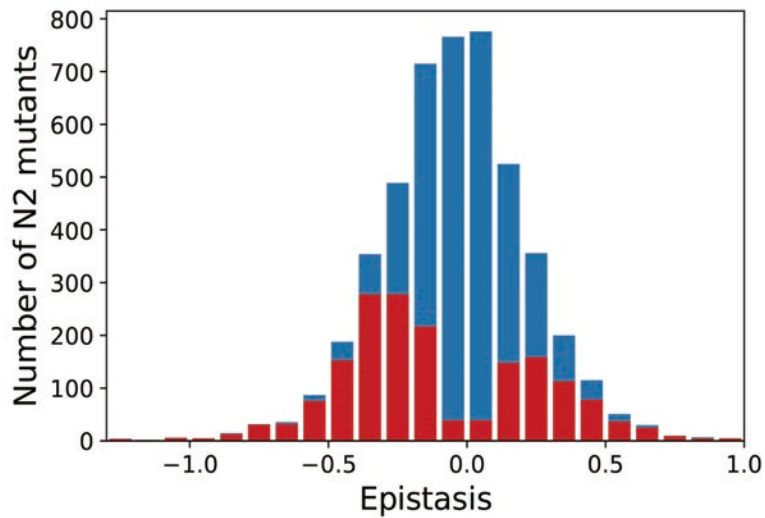

Supplement: msac086_Supplementary_Data [file msac086_supplementary_data.zip › Figure_S6.pdf]

**A**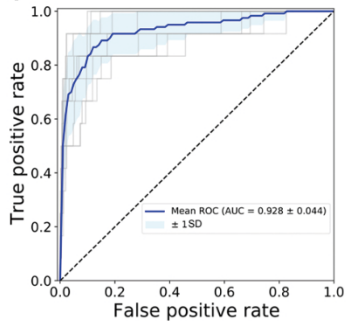**B**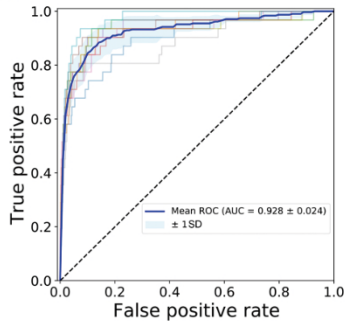**C**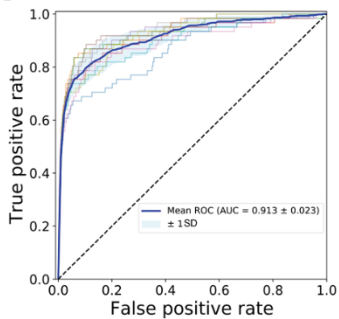

Supplement: msac086_Supplementary_Data [file msac086_supplementary_data.zip › Figure_S7.pdf]

**A**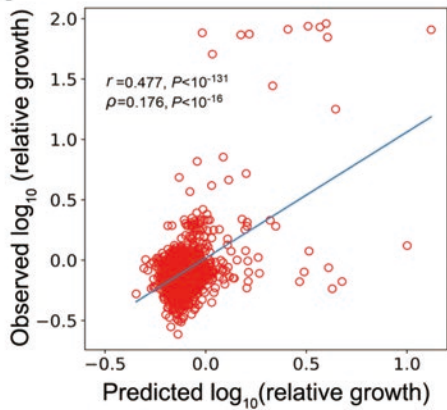**B**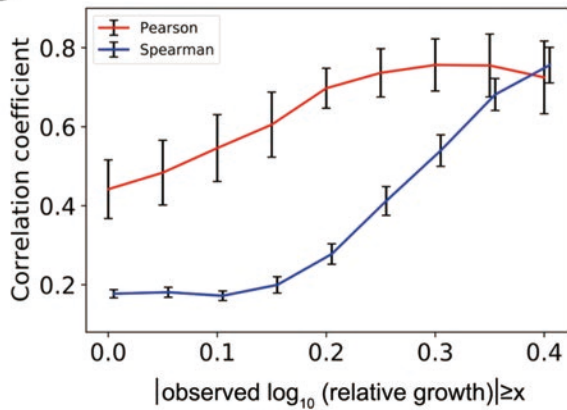

Supplement: msac086_Supplementary_Data [file msac086_supplementary_data.zip › Figure_S8.pdf]

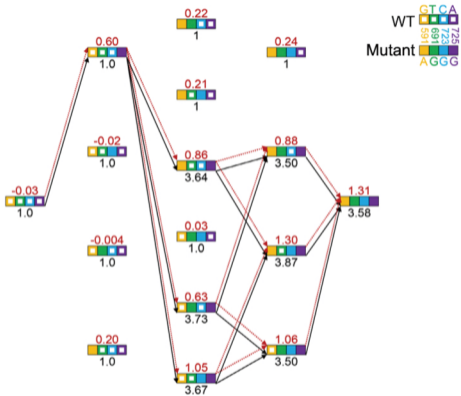

Supplement: msac086_Supplementary_Data [file msac086_supplementary_data.zip › Figure_S9.pdf]
